# Immune checkpoint crosstalk: CTLA-4/CD80 engagement as a predictor of anti-PD-1/PD-L1 therapy outcome in NSCLC

**DOI:** 10.3389/fimmu.2026.1783685

**Published:** 2026-07-16

**Authors:** Juan Gumuzio, James Miles, Nicole Quimi, Markel Rementeria, Cristina Cacho, Laura Camacho, Erica J. Geraedts, Kim van Elst, Fernando Aguirre, Peter J. Parker, Véronique Calleja

**Affiliations:** 1 HAWK Biosystems (formerly known as FASTBASE Solutions S.L.), Derio, Bizkaia, Spain; 2 Department of Pulmonology, Groene Hart Ziekenhuis, Gouda, Netherlands; 3 School of Cancer and Pharmaceutical Sciences, King’s College London, London, United Kingdom; 4 Francis Crick Institute, London, United Kingdom

**Keywords:** CTLA-4/CD80, FLIM–FRET, functional proteomics, immune oncology, interactomics, NSCLC, patient stratification, PD-1/PD-L1

## Abstract

**Background:**

CTLA-4/CD80 and PD-1/PD-L1 immune checkpoint pathways play critical roles in regulating T-cell activity within the tumor microenvironment (TME) of non-small cell lung cancer (NSCLC). While immune checkpoint inhibitors (ICIs) targeting PD-1/PD-L1 have improved survival outcomes, response rates vary, and predictive biomarkers’ alternative to PD-L1 expression are imperative. Our study aimed to identify PD-1/PD-L1 and CTLA-4/CD80 interaction states as enhanced predictive biomarkers for patient stratification to anti-PD-1/PD-L1 therapies in NSCLC.

**Methods:**

This study utilized the QF-Pro^®^ platform to quantify CTLA-4/CD80 and PD-1/PD-L1 interaction states in formalin-fixed paraffin-embedded (FFPE) samples from a cohort of 67 NSCLC patients treated with anti-PD-1/PD-L1 therapies. Corroborating prior findings, high PD-1/PD-L1 interaction, measured by FRET efficiency, predicts improved response rates via overall survival (OS) and progression-free survival (PFS).

**Results:**

Remarkably, we also identified high CTLA-4/CD80 interaction (FRET efficiency ≥6.41%) as correlating with improved OS and PFS in response to anti-PD-1/PD-L1 therapies. Patients with concurrently high PD-1/PD-L1 and CTLA-4/CD80 interactions exhibited the highest response rates, suggesting synergistic interplay between these checkpoints. In contrast, neither biomarker predicted response to chemotherapy in a separate cohort of 51 lung adenocarcinoma patients, confirming specificity for ICI therapy.

**Conclusions:**

These results highlight the potential of QF-Pro^®^ as a robust tool for stratifying NSCLC patients for ICI treatment and underscore the clinical relevance of CTLA-4/CD80 interaction as a predictive biomarker, paving the way for personalized immunotherapy strategies.

## Introduction

Lung cancer remains the leading cause of cancer-related mortality worldwide, accounting for 1.8 million deaths annually (1). Despite advances in targeted therapies and conventional treatment strategies, the prognosis for patients with advanced non-small cell lung cancer (NSCLC) remains poor, largely due to disease heterogeneity, late-stage diagnosis, and the development of therapeutic resistance (2). Over the past decade, immune checkpoint blockade has emerged as a transformative approach in cancer treatment, harnessing the body’s own immune system to mount an effective antitumor response. Among the most well-characterized immune checkpoint pathways are the cytotoxic T-lymphocyte-associated protein 4 (CTLA-4)/CD80 and programmed death-1/programmed death-ligand 1 (PD-1/PD-L1) axes. These pathways are critical regulators of immune homeostasis, functioning to prevent excessive immune activation and autoimmunity under normal physiological conditions. However, in the context of NSCLC and other solid tumors, their dysregulation enables immune evasion by suppressing effective antitumor immune responses (3).

Of the two aforementioned checkpoints, CTLA-4 is a homolog of the co-stimulatory receptor CD28, expressed on activated T-cells and regulatory T-cells (Tregs), and competes with CD28 to bind CD80 and CD86 on antigen-presenting cells (4). Unlike CD28 engagement, CTLA-4 binding transmits inhibitory signals that dampen early T-cell priming, serving as an essential checkpoint in immune regulation, and in NSCLC, contributes to tumor progression by limiting the expansion of tumor-reactive T-cells within the TME (5). While CTLA-4/CD80 primarily regulates early T-cell priming within lymphoid tissue, PD-1/PD-L1 signalling operates predominantly within peripheral tissues and the TME. PD-1 is expressed on activated T-cells and its ligand PD-L1 is frequently upregulated on tumor cells in response to inflammatory cytokines, particularly IFN-γ, enabling immune evasion by suppressing cytotoxic T-cell function (6). Engagement of PD-1 with PD-L1 recruits SHP-2, attenuating TCR signalling and driving T-cell exhaustion (7). Blockade of either axis has demonstrated clinical benefit in NSCLC; however, both ipilimumab-based CTLA-4 inhibition and PD-1/PD-L1 inhibitors are associated with incomplete response rates, resistance mechanisms, and in the case of CTLA-4 blockade, a higher burden of irAEs, underscoring the need for better patient stratification (8, 9).

Approved ICIs targeting PD-1 (pembrolizumab, nivolumab) or PD-L1 (atezolizumab, durvalumab) yield significant improvements in OS and PFS, however response rates remain low. Additionally, a substantial proportion of patients exhibit primary or acquired resistance, driven by mechanisms including alternative checkpoint upregulation, loss of antigen presentation, and an immunosuppressive TME, highlighting the need for better predictive biomarkers (10, 11). While PD-L1 expression is routinely used for patient stratification, its predictive value is generally poor and does not correlate with the *in situ* interaction state of PD-1/PD-L1 — the true target of these therapeutics. In a retrospective clinical study by our group, we previously quantified the interaction state of PD-1/PD-L1 within NSCLC biopsy samples and showed that above a threshold (2.13% FRET Efficiency), this interaction state was predictive of response to anti-PD-1/PD-L1 therapeutics (12). This previous study acted as a learning dataset upon which we could then validate the defined clinical cutoff value. However, whilst this work has been carried out in the field of PD-1/PD-L1, in the case of CTLA-4/CD80 therapeutics, no single direct predictive biomarker exists to stratify patients to receive ipilimumab (13). Critically, CD80 acts as a ligand for both CTLA-4 and PD-L1, with its competitive binding dynamics potentially influencing PD-1/PD-L1 engagement, this raises the question of whether these interaction biomarkers are mutually exclusive or functionally congruent (14).

To address this issue, we determined the engagement for both immune checkpoint complexes in NSCLC patients treated with PD-1/PD-L1 checkpoint blockade. The evidence indicates that these biomarkers significantly overlap and indeed patients who exhibit high interaction states of both checkpoints are seen to respond most effectively to immune checkpoint inhibition. Moreover, building on our earlier work, where we established a clinically relevant cutoff for PD-1/PD-L1 interaction states predictive of response to anti-PD-1/PD-L1 therapy (n=188), we demonstrate here the robustness of our technology by confirming a nearly identical threshold (2.06%) in the present cohort (n=67). Having established and validated these benchmarks, the focus of this study is to extend our approach to the CTLA-4 axis, characterizing its interaction states and their relevance for clinical response.

## Materials and methods

### Antibodies and reagents

Monoclonal antibodies mouse anti-PD-1, rabbit anti-PD-L1, and mouse anti-CTLA-4 were purchased from Abcam (catalogue numbers ab52587, ab205921, and ab19792, respectively). Polyclonal rabbit anti-CD80 was purchased from MyBioSource (catalogue number MBS2522916). AffiniPure F(ab’)_2_ fragment donkey anti-mouse IgG (H+L) and peroxidase AffiniPure F(ab’)_2_ fragment donkey anti-rabbit IgG (H+L) were purchased from Jackson Immuno Research (catalogue numbers 715-006–150 and 711-036-152, respectively). Pierce endogenous peroxidase suppressor, TSA SuperBoost kit, and a Prolong Glass antifade mount were purchased from Thermofisher Scientific (catalogue numbers 35000, B40925 and P36980 respectively). ATTO488 NHS ester, bovine serum albumin, and rhodamine B were purchased from Sigma Aldrich (catalogue numbers 41698-1MG-F, A2153-100G, and 234141-10G, respectively). ATTO488 NHS ester was conjugated to the AffiniPure F(ab’)_2_ fragment donkey anti-rabbit IgG (H+L) as described in Veeriah et al., 2014 (15).

### NSCLC cohort

Biopsies from NSCLC tumors were obtained during interventional radiology procedures or surgery. Samples and data from patients included in this study were provided by the Valdecilla Biobank (National Registry of Biobanks B. B.0000162) and Groene Hart Hospital (Gouda, Netherlands). All patients gave written informed consent for the use of their samples for research purposes. The Ethical and Scientific Committees form the Basque Country (CEIm-E) approved this study (CEIm-E PI2020200). The Medical Research Ethics Committee (MREC) of the BEBO Foundation for the Assessment of Ethics of Biomedical Research, Assen, declared that the Medical Research involving Human Subjects Act did not apply to this study and, therefore, no official approval was required by Dutch national law. The MREC of the BEBO Foundation is recognized by the Central Committee on Research Involving Human Subjects (CCMO) and accredited by the Dutch Association of MRECs (NVMETC). Valdecilla Biobank is integrated in the Platform ISCIII Biobanks and Biomodels (PT20/00067) and the samples were processed following standard operating procedures with the appropriate approval of the Ethics and Scientific Committees.

ICI-treated NSCLC cohort (n=67): FFPE biopsies were obtained from patients with histologically confirmed NSCLC (adenocarcinoma and squamous cell carcinoma) and samples obtained from primary tumours or metastases. Patients were treated with anti-PD-1/PD-L1 therapies (pembrolizumab, n=39; durvalumab, n=9; nivolumab, n=1; unspecified ICI, n=18) at the Valdecilla Biobank (Spain) and Groene Hart Hospital (Netherlands). Samples were determined as PD-L1 high (≥1%) or low (<1%) using the Roche VENTANA PD-L1 (SP263) assay except where indicated. Tumor Proportion Score (TPS) focuses on the percentage of viable tumor cells expressing PD-L1. Treatment was administered as monotherapy or in combination with chemotherapy, across first, second, and third lines. The cohort comprises predominantly advanced-stage (stage III–IV) disease, consistent with the clinical indication for ICI. Baseline clinical characteristics are detailed in **Supplementary Table 1**, including PD-L1 TPS, treatment line, and histological subtype.

The slides were labelled as donor-only and donor-acceptor, respectively (see below QF-Pro^®^ assay labelling).

### Lung adenocarcinoma microarray

Chemotherapy-treated lung adenocarcinoma TMA cohort (n=51): A tissue microarray (TMA) was obtained from the Cooperative Human Tissue Network (CHTN, funded by the National Cancer Institute) comprising 51 patients with lung adenocarcinoma treated with standard-of-care chemotherapy. This cohort is predominantly early-stage: stage IA (n=20, 40.8%), stage IB (n=9, 18.4%), stage IIA (n=10, 20.4%), stage IIB (n=6, 12.2%), stage IIIA (n=4, 8.2%), and stage IV (n=2, 4.1%). Two patients lacked overall survival data and one lacked PFS data. This cohort serves as an ICI-naïve specificity control.

Four slides with four consecutive slices of the TMA were analyzed following the same staining scheme as with NSCLC samples.

### QF-Pro^®^ assay principles

QF-Pro^®^ utilizes a two-site coincidence labelling assay to detect intercellular PD-1/PD-L1 interactions. Briefly, two primary monoclonal antibodies are used to detect PD-1 or CTLA-4 and PD-L1 or CD80 respectively. These antibodies are then labelled with F(ab’)_2_ fragments conjugated to the donor fluorophore ATTO488 (for PD-1 or CTLA-4 detection) and horseradish peroxidase (HRP) (for PD-L1 or CD80 detection). Tyramide signal amplification was used to label the F(ab’)_2_-HRP with the acceptor fluorophore Alexa-594. The conjugation of the fluorophores to F(ab’)_2_ fragments, which bind to the two primary antibodies, allows the quantification by FRET of protein–protein interactions as described previously by our group (12, 16). Unlike traditional FRET methods, the amplification of the acceptor fluorophore in QF-Pro^®^ increases the signal-to-noise ratio by four, allowing for robust FRET quantifications directly within fixed patient samples. For each patient condition, two samples are prepared: one labelled with only the donor primary and secondary antibodies, and the other labelled with both the donor and acceptor primary and secondary antibodies. Both samples are then excited using a 488 nm laser. In the first sample, which contains only the donor, the fluorescence lifetime of the donor is recorded. In the second sample, where both the donor and acceptor are present, the donor’s fluorescence lifetime is measured again. A reduction in donor lifetime in the second sample indicates resonance energy transfer (FRET) between the donor and acceptor. Since FRET only occurs when the donor and acceptor are within 1–10 nm of each other, this effect permits the quantification of receptor-ligand interactions.

### QF-Pro^®^ assay labelling

Four FFPE tissue sections per patient were mounted on separate glass microscope slides. This allowed for donor-only and donor-acceptor labelling to be carried out for all patient samples. Both checkpoints were analyzed per patient. Samples were prepared as previously described in Sanchez-Magraner et al., 2023 (12). Briefly, samples underwent antigen retrieval and dewaxing, blocking and incubation with both primary antibodies against either PD-1 and PD-L1 (antibody dilutions of 1:100 and 1:500 respectively) or CTLA-4 and CD80 (antibody dilutions of 1:200 and 1:500 respectively). Samples were then labelled with QF-Pro^®^ secondary reagents and tyramide signal amplification carried out prior to mounting of the samples. For TMA samples, the same staining protocol was followed.

### QF-Pro^®^ acquisitions

For QF-Pro^®^ acquisitions, a Nikon Ti2 Eclipse epifluorescence microscope is used. This is coupled to a multiple-frequency FLIM detector (PCO, Germany). The donor fluorophore (labelled to anti-PD-1) of the samples were excited using a 200mW 30MHz modulated 488nm diode laser (PCO, Germany). The laser underwent 50% modulation with a 100% modulation depth, resulting in an average optical power of 100mW. Acquisitions with the laser allowed for the simultaneous calculation of donor fluorophore lifetime and intensity (reporting on PD-1 or CTLA-4 expression). A metal-halide excitation lamp with a TRITC filter was used for the visualization of PD-L1 and CD80 expression (labelled with the Alexa-594 fluorophore). Samples were acquired using a Nikon CFI P-Apo DM 20X objective (N/A 0.75). The exposure time used was 800ms for lifetime acquisitions with an average optical power (post-modulation) of 100 mW. The exposure time used for acceptor intensity acquisitions was 60ms. The QF-Pro^®^ platform is coupled to a semi-automated high-throughput mapping and acquisition software. This allows a user to map positions of interest on a slide (x, y, and z coordinates) prior to their automatic and sequential acquisition. Phase lifetimes, average intensities, and lifetime images were automatically calculated. A decrease in donor lifetime (τD) in the presence of the acceptor fluorophore (τDA) is indicative of resonance energy transfer. FRET efficiency (E_ff_ %) values were calculated using the following equation, where τD and τDA are the lifetimes of the donor in the absence and presence of the acceptor, respectively:


$$E\text{ff }(\%)=[1-(\frac{\tau DA}{\tau D})]\times 100$$

### Statistical analysis

Data was tested for normality using the Shapiro-Wilks test, resulting in sufficient evidence of non-normality. Box-and-whisker plots were generated using GraphPad Prism 10. Here, the boxes represent the 1-99% range of the data and the whiskers represent the minimum and maximum values. GraphPad Prism 10 was also used to calculate Kaplan-Meier survival curves. For PD-1/PD-L1 interaction, NSCLC patients were ranked in order of their mean FRET efficiencies (interaction status) and split into two groups. Threshold for PD-1/PD-L1 interactions were derived from our prior study (Sanchez-Magraner et al., 2023). Here, this threshold was applied without modification using the cutoff value of 2.13%. For CTLA-4/CD80, the webtool, Cutoff Finder, was utilized to define objectively a cutoff point for survival analysis. The tool, described by Budczies et al., 2012, utilizes R scripts (17). The obtained cutoff value was 6.41%. Within the webtool, log-rank survival significance was utilized and determined the cutoff point at 6.41%, which separates the cohort in groups to be the lowest 60% and highest 40% of FRET Efficiencies. The log-rank (Mantel–Cox) test was carried out to determine significant differences between the groups. Kaplan-Meier curves were then plotted using GraphPad Prism 10. The Spearman’s r (r_s_) coefficient was also calculated using GraphPad Prism 10.

The probability for the patients at risk of experiencing the event was calculated as follows using GraphPad Prism 10, S(t)=S(t-1) x [N(t)-E(t)]/N(t).

## Results

### Quantification of CTLA-4/CD80 and PD-1/PD-L1 functional interaction in NSCLC tumor samples

**Figure 1:**
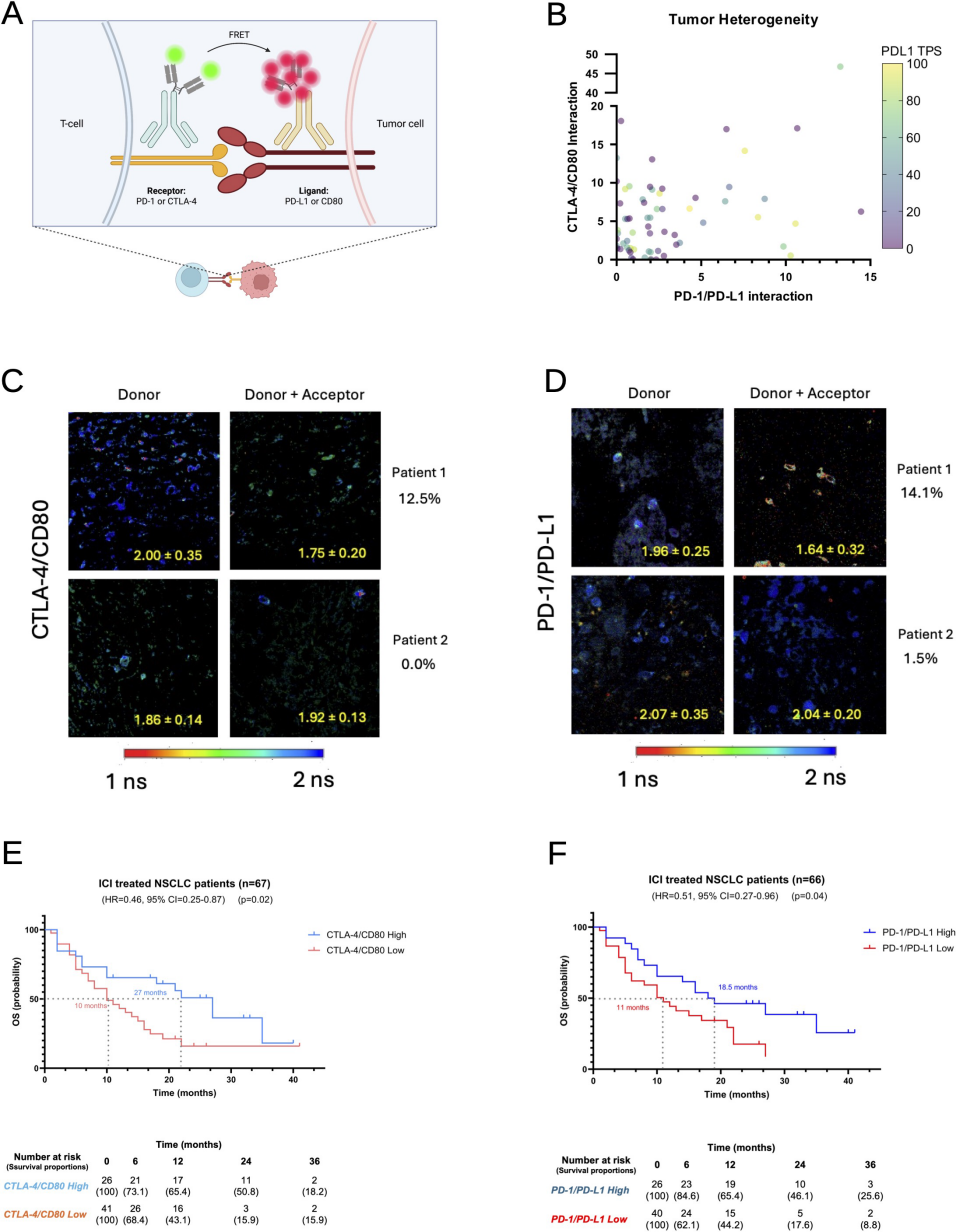
QF-Pro detects a high degree of inter- and intratumoral heterogeneity in PD-1/PDL1 and CTLA-4/CD80 interactions in NSCLC patients. **(A)** QF-Pro^®^ is a two-site immunofluorescence assay which utilizes FLIM–FRET to spatially quantify PD-1PD-L1 and CTLA-4/CD80 interactions at a 1-10nm resolution. The receptor/ligand pairs are targeted with primary antibodies which are in turn labelled with secondary F(ab’)2 fragments, the receptor antibodies conjugated to ATTO488 (donor) and those of the ligands conjugated to amplified Alexa594 (acceptor). **(B)** Correlation plot where each dot represents a patient’s sample, shows no correlation between the color-coded patients’ PD-L1 TPS scores and of either PD-1/PD-L1 or CTLA-4/CD80 interaction (FRET efficiency shown in x and y-axis). **(C)** Representative CTLA-4/CD80 FLIM maps showing donor-only (CTLA-4) and donor+acceptor (CTLA-4/CD80) conditions. Pseudo-color lifetime maps display per-pixel donor lifetime, with blue indicating longer lifetimes (~2 ns) and red shorter (~1 ns). Mean donor lifetimes are noted below for each map. A decrease in donor lifetime indicates FRET. The top row shows a patient with high interaction as determined by FRET efficiency (12.5%, lifetime drop from 2.00 ns to 1.75 ns). Bottom row shows a patient with low interaction as determined by FRET (0%). **(D)** Representative PD-1/PD-L1 maps with donor-only (PD-1) and donor+acceptor (PD-1/PD-L1). Top row shows a patient with high interaction as determined by FRET efficiency (14.1%). Bottom row shows a patient with low interaction as determined by FRET (1.5%). **(E)** The cutoff value applied to stratify patients based on CTLA-4/CD80 interaction was 6.41%. This grouping was determined on log-rank survival significance using *cutoff finder* webtool (17). Kaplan-Meier survival graph indicates that the patients with a higher FRET efficiency (light blue line) had a statistically significant higher OS (P = 0.02; HR = 0.46, 95% CI = 0.25-0.87) with a median survival of 27months compared to the remaining patients with a lower FRET efficiency (orange line) and a median survival time of only 10 months. **(F)** The cutoff value applied to stratified patients was 2.13127%, which separates the 40% of patients having a higher FRET efficiency versus the remaining 60% of patients with a lower FRET efficiency. This grouping was determined based on previous studies (17). Strikingly, the Kaplan-Meier survival graph indicates that the patients with a higher FRET efficiency (blue line) had a statistically significant higher OS (P = 0.03; HR = 0.51, 95% CI = 0.27-0.96) with a median survival of 18.5 months compared to the remaining patients with a lower FRET efficiency (red line) and a median survival time of only 11 months.

The interaction states of CTLA-4/CD80 and PD-1/PD-L1, were analyzed in 67 NSCLC patients using QF-Pro^®^ (**Figure 1A**). Within 66 patients, these interactions states were compared to routinely collected PD-L1 expression data (no PD-L1 TPS available for one patient). In one patient, PD-1/PD-L1 interaction state could not be calculated due to tissue sample degradation.

These interactions demonstrated inter-patient heterogeneity. The correlation plot presents PD-1/PD-L1 and CTLA-4/CD80 interactions for each patient (dots on the graph) and the color indicates the range of PD-L1 expression (TPS-PD-L1) for the same patients. The plot shows no correlation between the PD-L1 TPS scores and either of the interaction states (**Figure 1B**). Representative images of CTLA-4/CD80 and of PD-1/PD-L1 (FLIM maps) are shown in **Figures 1C, D** respectively. The images show the donor-only (CTLA-4 or PD-1) and the donor in presence of the acceptor (CTLA-4/CD80 or PD-1/PD-L1) for two representative patients. The top rows show patients with high interaction of the checkpoint as determined by a high FRET efficiency of 12.5% and 14.1% respectively. The bottom rows show patients with low interaction (FRET = 0% and 1.5% respectively). The pseudo-color lifetime maps display per-pixel donor lifetime as indicated in the scale bar (long lifetimes in blue and shorter lifetimes in green and red). A decrease in donor lifetime indicates FRET, which is presented as mean donor lifetimes in yellow on each map.

The mean FRET efficiency for CTLA-4/CD80 interaction was higher than that observed for PD-1/PD-L1, with an overall population average of 6.00% compared to 2.97%. Linear regression analysis was conducted to assess the correlation between both CTLA-4/CD80 and PD-1/PD-L1 interactions and PD-L1 TPS expression. No correlation was observed between CTLA-4/CD80 interaction and PD-L1 expression (Spearman’s correlation coefficient r_s_ = 0.001, P = 0.76) (**Supplementary Figure 1A**). The lack of correlation between PD-1/PD-L1 interaction and PD-L1 expression (r_s_ = 0.03, P = 0.17) was consistent with observations from previous studies (12, 16) (**Supplementary Figure 1B**). A weak positive correlation was observed between CTLA-4/CD80 interaction and PD-1/PD-L1 interaction across the full cohort (r_s_ = 0.1678, P<0.01) (**Supplementary Figure 1C**) suggesting a functional relationship for these immune checkpoint pathways in patients (see further outcome data below). Because FLIM–FRET detects molecular proximity within 1–10 nm, the assay can quantify interaction events occurring below the diffraction limit of light microscopy (18). This enables the detection of functional receptor–ligand engagement even when conventional expression scoring (e.g., TPS) is zero. To evaluate the clinical relevance of CTLA-4/CD80 and PD-1/PD-L1 functional interactions as predictive biomarkers in NSCLC, initial survival analyses were conducted based on mean FRET efficiency values for the individual markers. A threshold of 6.41% FRET efficiency was identified for CTLA-4/CD80 through correlation with OS. Patients classified within the CTLA-4/CD80 high group (≥6.41%) were found to have significantly longer OS compared to those in the low group (<6.41%) (median OS: 22 vs. 11 months; P = 0.03; HR = 0.53, 95% CI: 0.29–0.96) (**Figure 1E**). A favorable trend in PFS was also observed (P = 0.08; HR = 0.62, 95% CI: 0.36–1.07) (**Supplementary Figure 2A**). This predictive value was maintained across subgroups defined by treatment modality (monotherapy vs. chemo-ICI), line of treatment, and tumor histology. The strongest predictive performance was observed in first-line treatment (HR = 0.36, P < 0.001) and combination regimens (HR = 0.45, P = 0.03) (**Table 1**).

**Table 1:** Forest plot of OS (top) and PFS (bottom) showing the hazard ratio and 95% CI for each biomarker’s combinations analyzed in the different subgroups of patients based on the line of treatment (first and second line), treatment regime (monotherapy and in combination with chemotherapy) and tumor histology (adenocarcinoma and SCC).

Overall Survival																
Subgroup	PD-1/PD-L1 Interaction				CTLA-4/CD80 Interaction				PD-L1 (TPS ≥ 50%)				PD-L1 (TPS ≥ 1%)			
	*Lower*	*HR*	*Upper*	*P*	*Lower*	*HR*	*Upper*	*P*	*Lower*	*HR*	*Upper*	*P*	*Lower*	*HR*	*Upper*	*P*
**n=66**	0.27	0.48	0.96	0.03	0.24	0.53	0.87	0.02	0.36	0.68	1.27	0.21	0.34	0.68	1.27	0.22
**First Line (n=46)**	0.28	0.62	1.24	0.16	0.13	0.37	0.58	<0.001	0.30	0.58	1.28	0.20	0.30	0.68	1.37	0.24
**Second Line (n=20)**	0.12	0.40	1.40	0.16	0.32	1.15	3.89	0.85	0.14	0.54	2.06	0.20	0.16	0.55	2.03	0.38
**Monotherapy (n=29)**	0.21	0.43	1.45	0.22	0.21	0.59	1.48	0.24	0.36	0.99	2.76	0.99	0.17	0.75	2.94	0.64
**Combination (n=37)**	0.18	0.45	1.00	0.05	0.15	0.45	0.80	0.01	0.21	0.56	1.50	0.32	0.31	0.73	1.59	0.70
**Adenocarcinoma (n=39)**	0.14	0.43	0.88	0.02	0.21	0.59	1.09	0.09	0.34	0.75	1.78	0.56	0.41	0.94	2.06	0.86
**SCC (n=22)**	0.28	0.88	2.82	0.83	0.13	0.50	1.23	0.11	0.29	0.85	2.44	0.74	0.02	0.50	2.04	0.17
Progression Free Survival																
Subgroup	PD-1/PD-L1 Interaction				CTLA-4/CD80 Interaction				PD-L1 (TPS ≥ 50%)				PD-L1 (TPS ≥ 1%)			
	*Lower*	*HR*	*Upper*	*P*	*Lower*	*HR*	*Upper*	*P*	*Lower*	*HR*	*Upper*	*P*	*Lower*	*HR*	*Upper*	*P*
**n=66**	0.22	0.35	0.73	<0.01	0.32	0.62	1.03	0.06	0.34	0.68	1.27	0.57	0.45	0.71	1.95	0.2
**First Line (n=46)**	0.22	0.55	0.95	0.03	0.17	0.41	0.73	<0.01	0.32	0.66	1.28	0.20	0.24	0.64	1.17	0.12
**Second Line (n=20)**	0.12	0.42	1.00	0.05	0.55	0.55	5.80	0.33	0.38	0.95	3.90	0.74	0.29	0.48	3.24	0.96
**Monotherapy (n=29)**	0.23	0.58	1.58	0.31	0.21	0.73	1.48	0.24	0.42	0.90	3.02	0.81	0.13	0.62	2.67	0.50
**Combination (n=37)**	0.16	0.41	0.87	0.02	0.15	0.53	0.80	0.01	0.27	0.75	1.69	0.40	0.34	0.77	1.78	0.31
**Adenocarcinoma (n=39)**	0.11	0.35	0.59	<0.01	0.25	0.64	1.18	0.12	0.43	0.89	2.06	0.93	0.50	0.98	2.47	0.79
**SCC (n=22)**	0.24	0.50	2.04	0.51	0.23	0.55	1.87	0.43	0.34	0.95	2.73	0.95	0.00	0.52	0.64	0.05

• 95% CI (Lower–Upper) and P values are taken from the original figure (JPEG).

• HR, hazard ratio (point estimate). For all subgroups the HR is from the GraphPad analysis, EXCEPT 'Second Line' and 'SCC', where the HR was read from the diamond position in the figure (±0.05).

• TPS, tumour proportion score; SCC, squamous cell carcinoma; N/A, not available.

**Table:** 

n=66	First Line (n=47)	Second Line (n=19)	Monotherapy (n=28)	Combination (n=38)	Adenocarcinoma (n=40)	SCC (n=22)
0.37	0.294	0.1431	0.3664	0.2099	0.3348	0.2944
0.68	0.5828	0.5432	0.9921	0.5605	0.7491	0.8407
1.24	1.156	2.06	2.68	1.497	1.676	2.401

For PD-1/PD-L1, a FRET efficiency threshold of 2.09% was determined to provide optimal stratification of treatment outcomes in this cohort, consistent with a previously reported cutoff of 2.13% identified in an independent study from our group involving a cohort of 188 patients (12). To validate this threshold, patients were stratified using the 2.13% cutoff. Significantly improved survival outcomes were observed in patients with higher PD-1/PD-L1 interaction (≥2.13%), with a median OS of 20 months compared to 10 months in the low-interaction group (P 0.01; HR = 0.47, 95% CI: 0.26–0.88) (**Figure 1F**), and a median PFS of 14.5 vs. 4 months, respectively (P < 0.01; HR = 0.49, 95% CI: 0.28–0.86) (**Supplementary Figure 2B**). In contrast, stratification based on PD-L1 tumor proportion score (TPS) (≥1% vs. <1%) did not yield statistically significant differences in OS or PFS (**Supplementary Figure 3**), underscoring the superior predictive performance of the FRET-based interaction measurements. Subgroup analysis indicated that the predictive value of PD-1/PD-L1 interaction was particularly strong in adenocarcinoma cases (OS: P = 0.02; PFS: P < 0.01), whereas no significant stratification was observed in squamous cell carcinomas despite a trend seen in PFS (**Supplementary Figure 4**).

Taken together, these findings suggest that the direct quantification of immune checkpoint engagement in tumor tissue—via FRET-based assessment of CTLA-4/CD80 and PD-1/PD-L1 interactions—provides a robust and biologically relevant approach to predict response to immune checkpoint inhibitors in NSCLC. Unlike conventional expression-based markers, these functional biomarkers capture the dynamic immunoregulatory state of the TME, offering enhanced prognostic and stratification capabilities.

### PD-1/PD-L1 and CTLA-4/CD80 interactions in combination select a subset of patients with higher response to immunotherapy treatment

**Figure 2:**
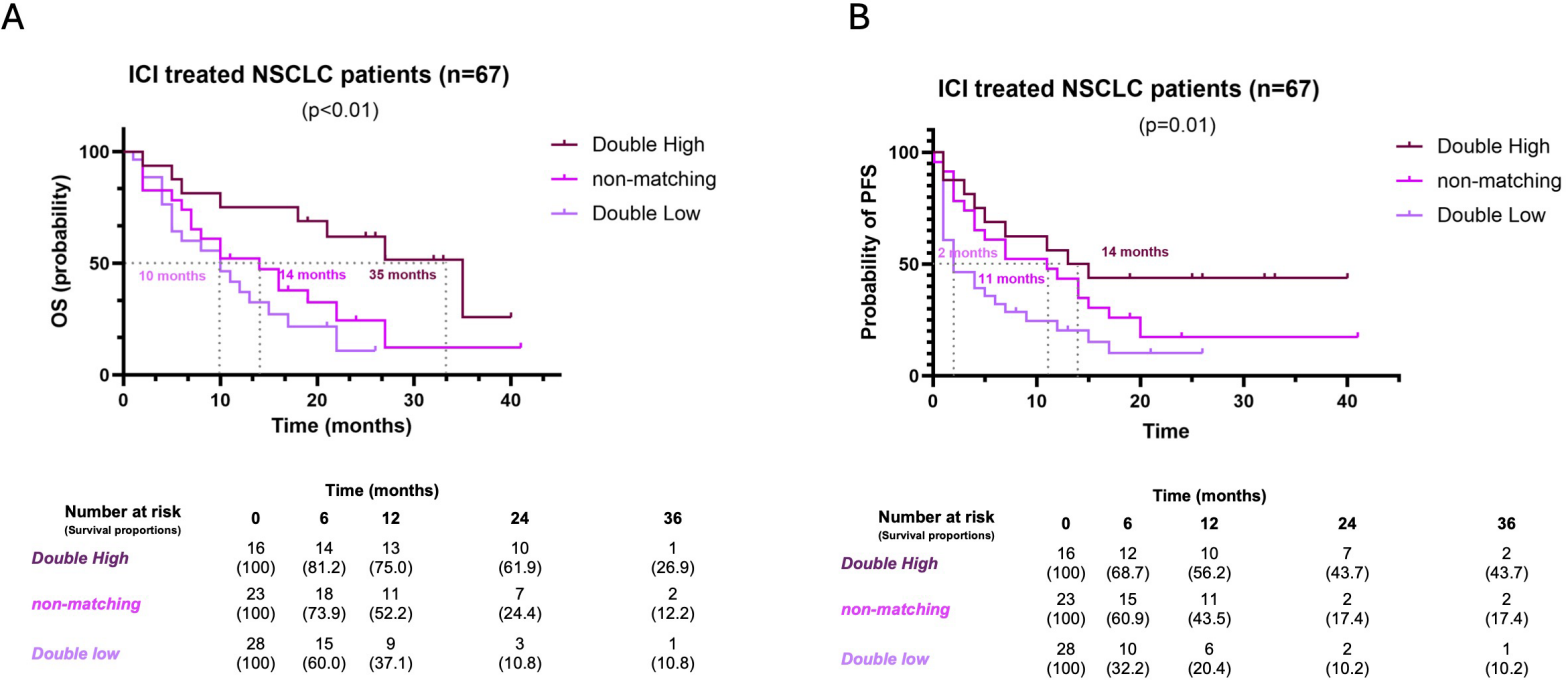
Combination of high PD-1/PD-L1 and high CTLA-4/CD80 interaction identifies a subset of NSCLC patients that better respond to ICI treatment. The OS **(A)** and PFS **(B)** of each patient was analyzed according to their classification as double high (high PD-1/PD-L1 and high CTLA-4/CD80 interaction), double low (low PD-1/PD-L1 and low CTLA-4/CD80 interaction) and non-matching (either one of the interactions is high), based on their FRET efficiency for both biomarkers. The tables present the survival probability **(A)** or the probability of progression **(B)** for the patients at each time point of the study (in months). The number of patients at risk is indicated for each immune checkpoint status (double high, non-matching, double low) and the probability to experiencing the event at a given time point is shown in brackets.

We investigated the possibility that combining both checkpoint biomarkers might better predict a response to ICI treatment. Patients were classified into three different subgroups based on their level of PD-1/PD-L1 and CTLA-4/CD80 interaction. Patients with high levels of both CTLA-4/CD80 and PD-1/PD-L1 interaction were classified as ‘*double high’*, patients with low levels of PD-1/PD-L1 and CTLA-4/CD80 interaction as ‘*double low’* and the rest as ‘*non-matching’*. When analyzing the OS based on this sub-group classification (**Figure 2A**), median OS was 35 months for the ‘*double high’* subpopulation, 14 months for the *‘non-matching’* patients and 10 months for the *‘double low’* sub-group. At 12 months, the ‘*double high’* sub-population exhibited an OS survival probability of 75.0% which dropped to 61.9% at 24 months. By contrast, the ‘*non-matching’* group shows a lower 12- and 24-month OS survival probability of 52.2% and 24.4% respectively, which drastically decreased to 37.1% and 10.8% at 12 and 24 months in the ‘*double low*’ subgroup.

For PFS (**Figure 2B**), at 6 months, the ‘*double high’* group maintained a PFS survival probability of 68.7%, while the ‘*non-matching’* and ‘*double low’* groups declined to 60.9% and 32.2%, respectively. By 12 months, disease progression was evident, with 56.2% of ‘*double high’* patients remaining progression-free, compared to 43.5% in the ‘*non-matching’* and 20.4% in the ‘*double low’* groups. At 24 months, PFS survival probability in the ‘*non-matching’* group was 17.4%, while the ‘*double low’* group had dropped below 10.2% by 21 months, indicating a poor response. The ‘*double high’* cohort maintained a PFS survival probability of 43.7% up to 40 months, indicating a sustained benefit.

### Neither CTLA-4/CD80 nor PD-1/PD-L1 interaction, alone or combined, predict response to chemotherapy in a cohort of lung adenocarcinoma patients

To assess the specificity of CTLA-4/CD80 and PD-1/PD-L1 interaction as a biomarker for response to ICI treatment, 51 samples from lung adenocarcinoma patients treated with standard of care (SOC) chemotherapy were analyzed. The TMA cohort comprised 51 patients diagnosed with lung adenocarcinoma, stratified according to tumor stage based on the TNM classification. Two of the patients lacked OS clinical outcome information and one PFS clinical outcome information. Early-stage disease (stage I–II) was predominant, with 20 patients classified as stage IA (40.8%), 9 as stage IB (18.4%), 10 as stage IIA (20.4%), and 6 as stage IIB (12.2%). A smaller subset presented with more advanced disease, including 4 patients with stage IIIA (8.2%) and 2 patients with stage IV disease (4.1%). This distribution reflects a patient population primarily composed of early-stage lung adenocarcinoma cases, with a minority of individuals exhibiting locally advanced or metastatic disease.

The mean FRET values obtained for CTLA-4/CD80 interaction closely resembled those observed in a cohort of 67 NSCLC patients treated with immunotherapy, although inter-patient variability was lower (5.31% ± 3.06 SD vs. 6.00% ± 6.31 SD).

**Figure 3:**
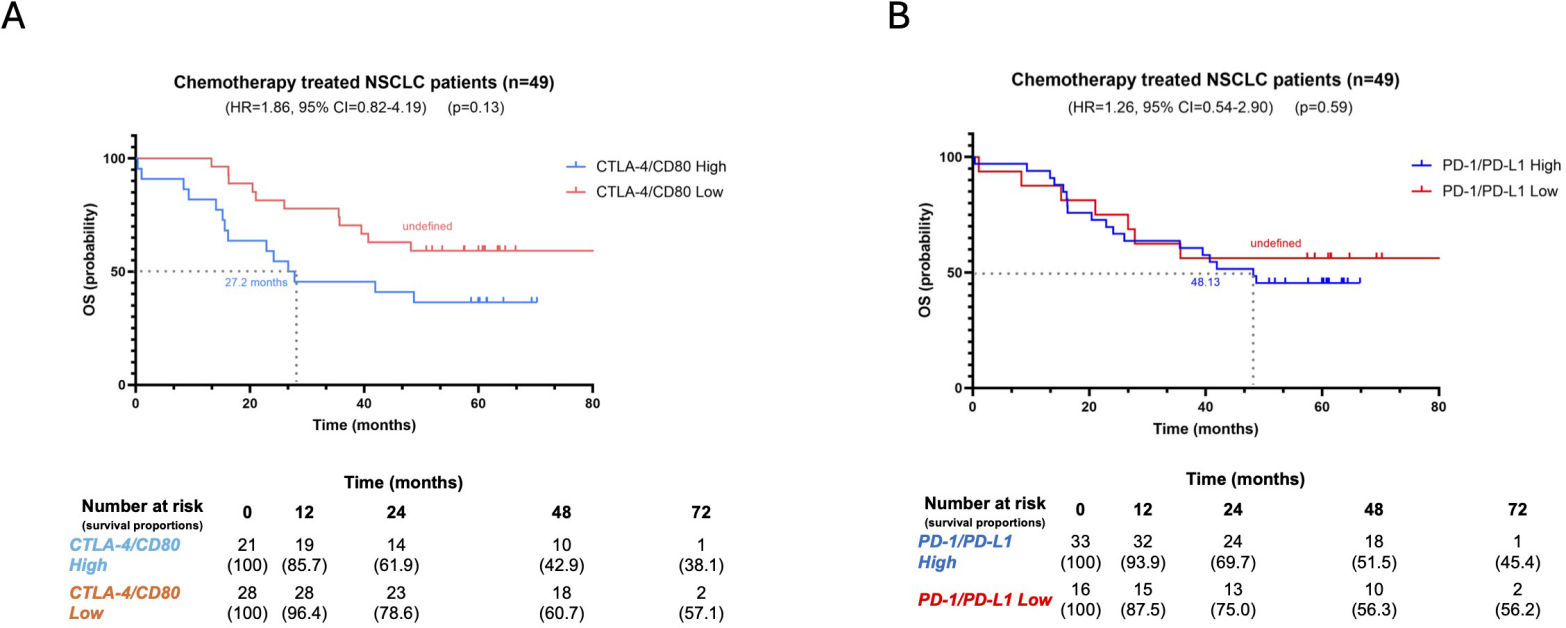
Neither CTLA-4/CD80 nor PD-1/PD-L1 interaction status is predictive of response to chemotherapy treatment in lung adenocarcinoma patients (n=49). **(A)** The OS of each patient was analyzed according to the calculated FRET efficiency. For CTLA-4/CD80 interaction, the cutoff value applied to stratified patients was 6.41%. Kaplan-Meier survival graph indicates significant differences in OS between patients with a higher FRET efficiency (light blue line) and patients with lower FRET efficiency (orange line) (High FRET efficiency vs Low FRET efficiency median OS 27.2 months vs undefined; P = 0.13; HR = 1.86; 95% CI = 0.82-4.19). **(B)** For PD-1/PD-L1 interaction, the cutoff value applied to stratified patients was 2.13%. This grouping was determined based on previous studies (17). Kaplan-Meier survival graph indicates no significant differences in OS between patients with a higher FRET efficiency (blue line) and patients with lower FRET efficiency (red line). High FRET efficiency vs Low FRET efficiency median OS undefined vs 48.13 months (P = 0.59; HR = 1.26; 95% CI = 0.54-2.90).

We then evaluated the predictive power of CTLA-4/CD80 interaction for chemotherapy response using Kaplan-Meier analysis, applying a threshold of 6.41% to stratify the patient cohort into CTLA-4/CD80 high and CTLA-4/CD80 low subpopulations. Interestingly, the trend observed in these patients was the opposite of that seen in those treated with immunotherapy. Patients in the CTLA-4/CD80 low group, while not reaching significance, exhibited a better response to treatment compared to those in the CTLA-4/CD80 high group, with median OS values of more than 50 months vs 27.2 months, respectively (P = 0.13; HR: 1.86; 95% CI [0.82–4.19]) (**Figure 3A**). The analysis of treatment response based on PFS demonstrated a comparable trend, with patients in the CTLA-4/CD80 low subgroup exhibiting a longer median PFS than those in the CTLA-4/CD80 high subgroup (median PFS 35.5 months vs 23.0 months) (**Supplementary Figure 3A**).

For PD-1/PD-L1 interaction quantification, stratification by FRET efficiency showed no significant OS differences (median undefined vs. 48.13 months, P = 0.59; HR = 1.26, 95% CI = 0.54-2.90) (**Figure 3B**) or PFS differences (median undefined vs. 25.7 months, P = 0.17; HR = 1.69, 95% CI = 0.80-3.54) between high and low FRET efficiency groups (**Supplementary Figure S3B**). Notably, while not reaching significance, we observed a worse median PFS trend for the patients treated with chemotherapy with a high PD-1/PD-L1 interaction. Similarly, stratification by PD-L1 TPS (≥1% vs. <1%) did not yield significant differences in OS (P = 0.49; HR = 0.76, 95% CI = 0.34-1.68) nor PFS (P = 0.78; HR = 0.90, 95% CI = 0.44-1.85) (**Supplementary Figure 4**). These results suggest that PD-1/PD-L1 interaction, as measured by FRET efficiency, lacks predictive value for survival in chemotherapy-treated adenocarcinoma patients.

**Figure 4:**
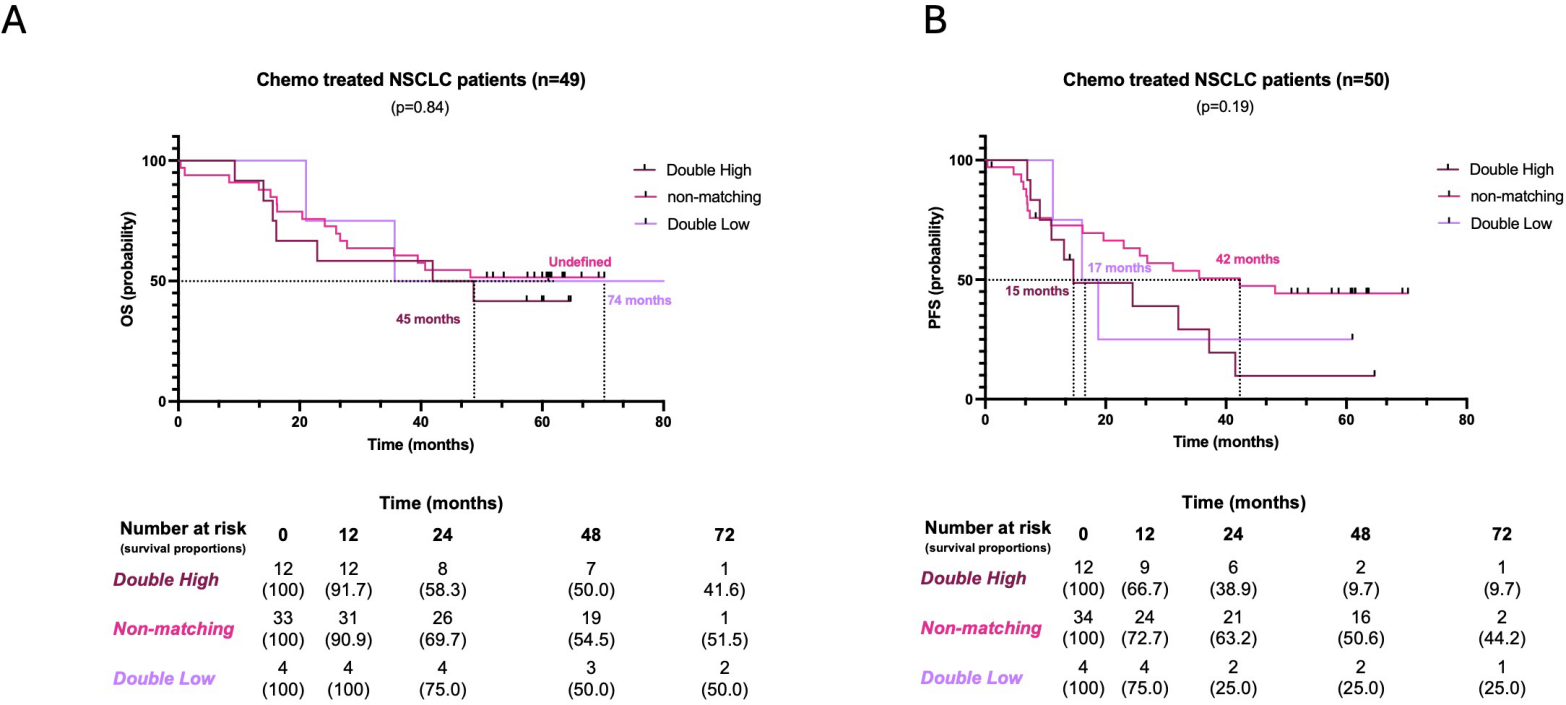
The combination of CTLA-4/CD80 and PD-1/PD-L1 interaction status is not predictive of response to chemotherapy treatment in lung adenocarcinoma patients (n=49). The OS **(A)** and PFS **(B)** of each patient was analyzed according to their classification as *double high* (high PD-1/PD-L1 and high CTLA-4/CD80 interaction), *double low* (low PD-1/PD-L1 and low CTLA-4/CD80 interaction) and *non-matching*, based on their FRET efficiency for both biomarkers.

Subsequently, the combined predictive value of CTLA-4/CD80 and PD-1/PD-L1 interaction for chemotherapy treatment response was evaluated. Patients were stratified based on the interaction levels of PD-1/PD-L1 and CTLA-4/CD80 into ‘*double high’*, ‘*double low’*, and ‘*non-matching’* subgroups. This stratification did not reveal statistically significant differences in treatment response as measured by OS (P = 0.89). Interestingly, the lowest median OS was observed in the *‘double high’* group (45 months), followed by the ‘*double low*’ group (74 months). Median OS was not reached in the ‘*non-matching’* group (**Figure 4A**). In terms of PFS, patients in the ‘*non-matching’* group exhibited better outcomes, with a median PFS of 42 months, whereas both the ‘*double high’* and ‘*double low’* groups showed similar trends with median PFS values of 15 and 17 months, respectively (P = 0.19) (**Figure 4B**).

## Discussion

The identification of functional biomarkers that provide mechanistic insights into therapeutic response remains a critical area of investigation for clinical implementation. In the context of immune checkpoint blockade therapies, the limitations of expression-based biomarkers, such as PD-L1 immunohistochemistry, have been well-documented (19). Our previous work demonstrated that *in situ* quantification of PD-1/PD-L1 interaction is a promising predictive biomarker of response to PD-1/PD-L1 blockade immunotherapy in patients with advanced NSCLC. Recent studies have further validated the predictive value of this interaction in NSCLC (20); however, for its clinical implementation, it is essential to establish robust, reproducible, and reliable quantification methods that are compatible with routine clinical workflows. In this study, we have evaluated in routinely collected patient biopsies the predictive power of measuring CTLA-4/CD80 interaction *in situ* to observe and predict response to anti-PD-1/PD-L1 therapies within 67 NSCLC. There was found to be a correlation between PD-1/PD-L1 high and CTLA-4/CD80 high engaged patients, which translated into the finding that combined double high readouts were highly significant predictors of response to ICI treatments. Correlating with this, we found that patients with just a high CTLA-4/CD80 interaction showed a significant survival benefit from ICI treatment. To our knowledge, the measurement of high CTLA-4/CD80 checkpoint engagement and prediction of a favorable response to PD-1/PD-L1 blockade therapies had not yet been reported. It should be noted that, whilst the predictive value of PD-1/PD-L1 has been confirmed within an independent patient cohort, this study served as a learning cohort for CTLA-4/CD80. Therefore, a future independent study assessing CTLA-4CD80 as a predictive biomarker will be required for full biomarker validation.

One interesting observation was the detection of interaction states in patients scored with ≤1% TPS (**Supplementary Figure 1**). Our findings underscore that interaction scoring is not constrained by the diffraction limit of conventional light microscopy. Whereas immunohistochemistry provides a morphological estimate of ligand expression at the cellular or regional level, FLIM–FRET directly measures nanoscale proximity between receptor and ligand pairs. This allows biologically relevant engagement events to be quantified even when tumor cell PD-L1 expression is scored as absent by TPS, explaining the presence of interaction states in TPS-0 tumors. This may also explain findings from other studies where response to anti-PD-1/PD-L1 interaction states were observed in patients with a PD-L1 TPS score of ≤1% (21, 22).

Whilst PD-L1 (and other ligand expression scores) often lack a high dynamic range, QF-Pro^®^ is also capable of detecting heterogeneity in interaction states. Our analysis revealed notable inter- and intra-patient variability in the measured CTLA-4/CD80 interaction states across NSCLC samples. This variability may reflect differences in the spatial distribution of the interaction within the tumor microenvironment, potentially influenced by factors such as immune cell infiltration, tumor architecture, or local expression patterns of CD80 and CTLA-4. These findings underscore the complexity of immune checkpoint dynamics and suggest that a single-region biopsy may not fully capture the heterogeneity of the interaction across the tumor. Future studies should aim to explore this spatial variability more comprehensively, possibly through multi-region sampling, to better understand its impact on treatment prediction and clinical outcomes.

In this study, we also further validated the individual PD-1/PD-L1 interaction as a predictive biomarker of response to immune checkpoint inhibitors in NSCLC patients, employing a previously established cutoff value derived from a cohort of 188 ICI-treated patients (12). The blinded analysis of the cohort of 67 NSCLC patients, using the same analytical platform, reagents, acquisition and analysis criteria (albeit with different operators), yielded highly consistent results with those observed in our original 2023 study. The similarity in the distribution of PD-1/PD-L1 interaction values across cohorts further supports the analytical robustness of the QF-Pro^®^ assay. Furthermore, applying the previously defined cutoff value to stratify the cohort into PD-1/PD-L1 high and PD-1/PD-L1 low subgroups, we observed that patients classified as PD-1/PD-L1 high exhibited superior treatment responses compared to those in the PD-1/PD-L1 low group. This trend replicates findings from the original study, reinforcing the potential of PD-1/PD-L1 interaction as a clinically relevant biomarker for predicting response to ICIs. Notably, no correlation was detected between FRET efficiency (PD-1/PD-L1 interaction) and PD-L1 expression levels assessed by conventional immunohistochemistry (SP263 Ventana Roche). This lack of correlation was observed both when PD-L1 TPS ≥1% and TPS ≥50% were used (**Supplementary Table 1**). This observation is consistent with our prior findings and aligns with reports from other solid malignancies, suggesting that PD-1/PD-L1 interaction may provide distinct biological insights beyond PD-L1 expression alone. Although both adenocarcinoma and squamous NSCLC benefit from ICI treatment, histology-specific analysis revealed significant predictive value of high PD-1/PD-L1 interaction only in adenocarcinoma, not in SCC. This is somewhat unexpected given the higher immunogenicity of SCC and its generally favorable response to ICIs in clinical settings. However, the very small number of high PD-1/PD-L1 cases in the SCC cohort (n=6) likely limited statistical power, despite a trend toward improved PFS. Further characterization of molecular features (e.g., mutation status, TMB) will be important to better understand these findings.

Critically, the QF-Pro^®^ approach employs Fluorescence Lifetime Imaging Microscopy (FLIM) to determine interaction states via time-resolved FLIM-FRET. As FLIM quantifies the donor fluorophore (ATTO488, labelling PD-1) lifetime in the presence and absence of the acceptor (Alexa594, labelling PD-L1), the measurement is independent of fluorophore concentration and thus of PD-1 expression levels. This makes the assay inherently robust to variability in fluorochrome excitation and instrument settings, unlike intensity-based FRET methods. Moreover, repeat measurements performed by multiple independent operators yielded consistent results, underscoring the reliability and reproducibility of the assay. In principle, this approach is reproducible across any wide-field FLIM system, provided it is appropriately tuned for ATTO488 donor lifetime. Because FRET efficiency is determined from the relative difference between donor-only and donor-acceptor lifetimes, small variations in absolute lifetime measurements between devices would be normalized. As a result, the calculated interaction scores remain consistent across instruments.

The specificity of CTLA-4/CD80 and PD-1/PD-L1 interaction as a predictive biomarker for ICI therapy, rather than a general prognostic biomarker for NSCLC, was further supported by the analysis of adenocarcinoma patient samples treated with chemotherapy. The lack of association between CTLA-4/CD80 and PD-1/PD-L1 interaction levels and chemotherapy response reinforces the biomarker’s predictive specificity for immune checkpoint blockade. However, some limitations should be acknowledged. The chemotherapy-treated cohort represents a smaller subset of patients, and tissue microarrays provide only a limited representation of possible intratumoral heterogeneity. Moreover, early-stage tumors (I–II) were overrepresented in the chemotherapy-treated cohort, whereas the ICI-treated cohort included a higher proportion of advanced-stage tumors (III–IV). This difference in stage distribution between the cohorts may partly explain the generally longer OS observed in the chemotherapy-treated group. Although the absence of an association with chemotherapy in this cohort appears robust, further validation in a larger and more representative patient population will be required to confirm the specificity of these biomarkers for patients treated with ICI. Additionally, the use of this cohort supports the specificity of the amplified FRET signals observed with QF-Pro^®^. The lack of correlation within this cohort serves as a functional negative control for non-specific signal contributions. Were bystander FRET (arising from proximity of fluorophores independent of true protein-protein interaction) contributing meaningfully to the measured signal, one would expect randomly distributed FRET efficiency values to produce spurious associations across both cohorts equally. The absence of any such association in the chemotherapy cohort therefore provides empirical evidence that the interaction scores reflect genuine receptor-ligand engagement rather than non-specific fluorophore proximity.

The clear stratification of patients’ response to ICI treatment achieved with the combined biomarkers readout likely reflects the complexity of immune checkpoint regulation, which arises from the interplay of multiple co-existing immunological and tumor-intrinsic factors. First, the interaction of CTLA-4 with its ligand CD80 can modulate the availability of CD80 for binding to PD-L1, thereby directly influencing the inhibitor-targeted PD-1/PD-L1 axis. In addition, recent studies have shown that CTLA-4 can capture and internalize CD80 from antigen-presenting cells through a process known as transendocytosis (23) disrupting its cis-heterodimerization with PD-L1 and increasing the availability of PD-L1 to engage with PD-1 on T-cells. Consequently, in the context of PD-1/PD-L1 blockade, prior engagement of CTLA-4 with CD80 could shift the balance of checkpoint interactions and influence PD-L1-mediated inhibition of the checkpoint, potentially enhancing the therapeutic efficacy of PD-1/PD-L1 inhibitors. Additionally, PD-L1/CD80 interaction has been shown to inhibit both PD-1 and CTLA-4 -mediated checkpoint pathways while preserving CD80 activation of the co-stimulatory receptor CD28 (24). Together, this suggests that the balance of these interactions is crucial in modulating immune responses and that these pathways may act in series, rather than in parallel to suppress immune surveillance. Therefore, the status of CTLA-4/CD80 interaction could reflect a TME more susceptible to PD-1/PD-L1 blockade, as the disruption of inhibitory signals may favor T cell activation and anti-tumor immunity. An additional factor that may explain the above finding is the distinct role of the two checkpoints during different phases of immune system activation. CTLA-4/CD80 interaction is often upregulated following the initial priming of T-cells (25). Thus, increased engagement of this checkpoint functions as a regulatory mechanism to limit the response associated with increased T-cell activation and expansion (26). However, after migration to the TME and under condition of prolonged antigen exposure, these tumor infiltrating lymphocytes may exhibit chronic activation, resulting in a local suppression of the immune response through PD-1/PD-L1 interaction (27).

The identification of high-responder patients to anti-PD-1/PD-L1 therapy through dual assessment of PD-1/PD-L1 and CTLA-4/CD80 interactions suggests that, in patients exhibiting high levels of both checkpoints (“double-high”) monotherapy, rather than combination therapy with ipilimumab (anti-CTLA-4), may achieve maximal response while minimizing immune-related adverse events (irAEs), potentially offering a safer and more effective treatment strategy. Whilst this study assessed response rates to anti-PD-1/PD-L1 therapies, this paves the way for the generation of functional immune checkpoint signatures for patients. This would allow for advances in personalized medicines, by functionally stratifying patients to receive the most appropriate immunotherapies, with a focus on emerging therapeutics targeting other immune checkpoints like TIGIT/CD155, LAG3/MHCII and TIM3/Gal9 (28). Furthermore, this would increase response rates to these expensive therapies whilst minimizing the undesired immune-related adverse effects, thus increasing patient quality of life whilst simultaneously reducing healthcare cost burdens. While future studies can be used to apply QF-Pro^®^ to other biomarkers and immunogenic tumors, it would also be interesting to compare the predictive value of receptor-ligand interaction on treatment response vs other assay panels. For example, whilst CD8 infiltration data and genetic assay results (such as Ayers’ 18-gene T-effector signature) were not available in this cohort, integrating these markers in future studies could provide complementary insights into immune activity within the TME. However, it is important to recognize that gene expression levels do not always correlate directly with transcriptional activity and protein abundance, and even then, protein expression often fails to correlate with functional engagement. Therefore, these genetic screens may fail to capture the dynamic receptor–ligand interactions measured by our assay. Moreover, it is worth noting that the FRET measurements reported here capture checkpoint engagement at the level of bulk tissue, providing a powerful and clinically actionable readout without the need for complex multiplexed panels. Future studies incorporating co-staining with lineage markers such as CD3, CD68, and cytokeratins alongside the FRET pairs will offer the opportunity to further dissect the cellular origin of these signals and more precisely map the checkpoint crosstalk to defined tissue compartments, adding an additional layer of biological resolution to this assay.

In summary, we have demonstrated that combined PD-1/PD-L1 and CTLA-4/CD80 engagement predicts the most effective responses to PD-1/PD-L1 interventions. In so doing, we have further validated the predictive power of IC engagement as a basis for stratification to ICI. While this study also reconfirms the clinical cutoff value of PD-1/PD-L1 established in our earlier work, its principal novelty lies in extending this framework to CTLA-4 interactions. Prospective validation of CTLA-4/CD80 thresholds in independent cohorts will be an essential next step, but our findings provide the first evidence that CTLA-4 engagement may offer an additional and complementary dimension for patient selection in immunotherapy.

## Data Availability

The datasets presented in this article are not readily available because the raw imaging data and clinical datasets are not publicly shared to protect patient confidentiality under GDPR/Ethics protocols and involve proprietary QF-Pro® FRET-FLIM processing algorithms. Anonymized quantitative data can be provided by the corresponding author upon reasonable request. Requests to access the datasets should be directed to veronique.calleja@hawkbiosystems.com.
