# Supplementary figures and images for: Immune checkpoint crosstalk: CTLA-4/CD80 engagement as a predictor of anti-PD-1/PD-L1 therapy outcome in NSCLC
Source: Front Immunol. 2026 Jul 16;17:1783685. doi: 10.3389/fimmu.2026.1783685 (PMC13422567; doi:10.3389/fimmu.2026.1783685)

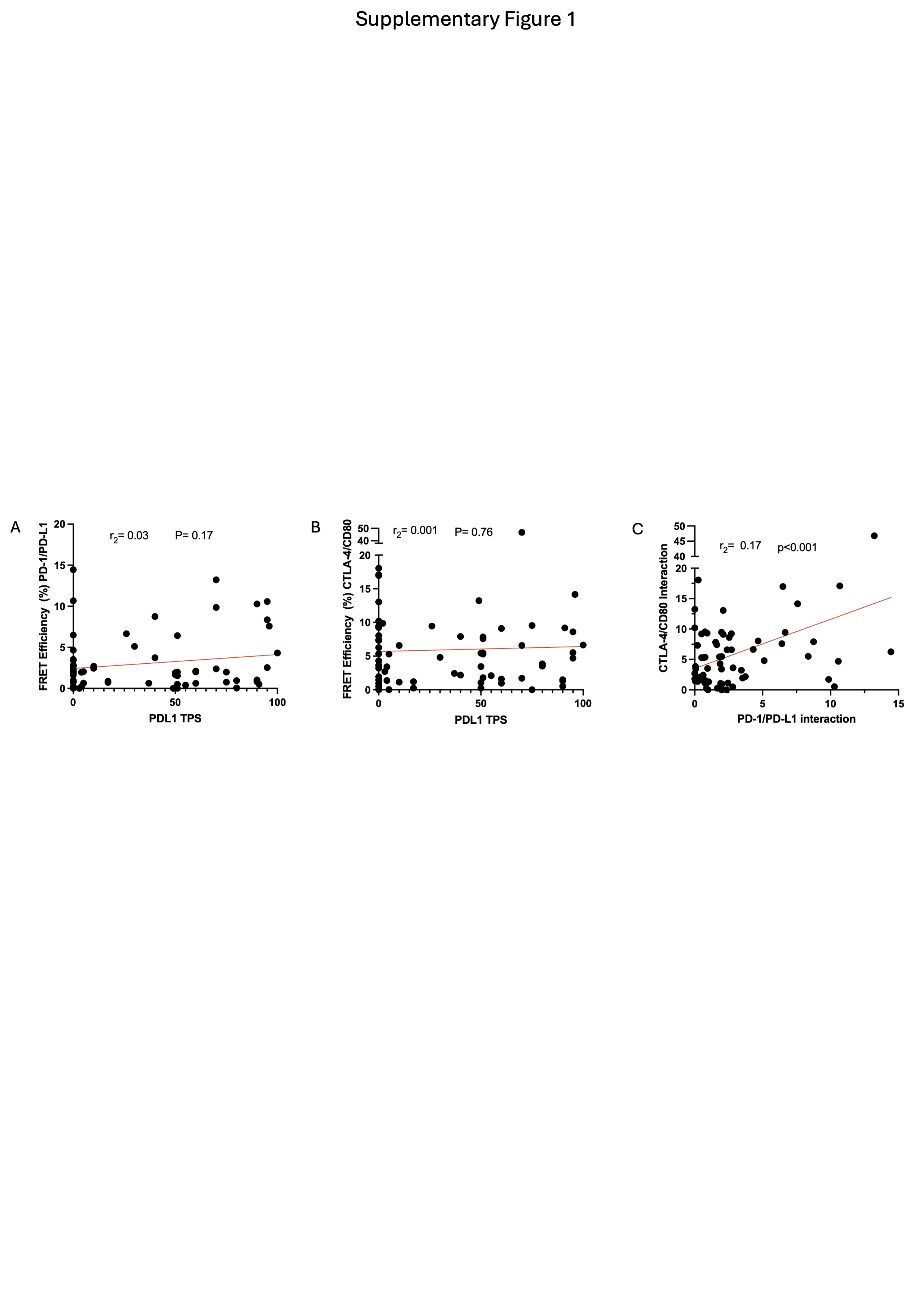

Supplement: Supplementary file 1 [file Image1.jpeg]

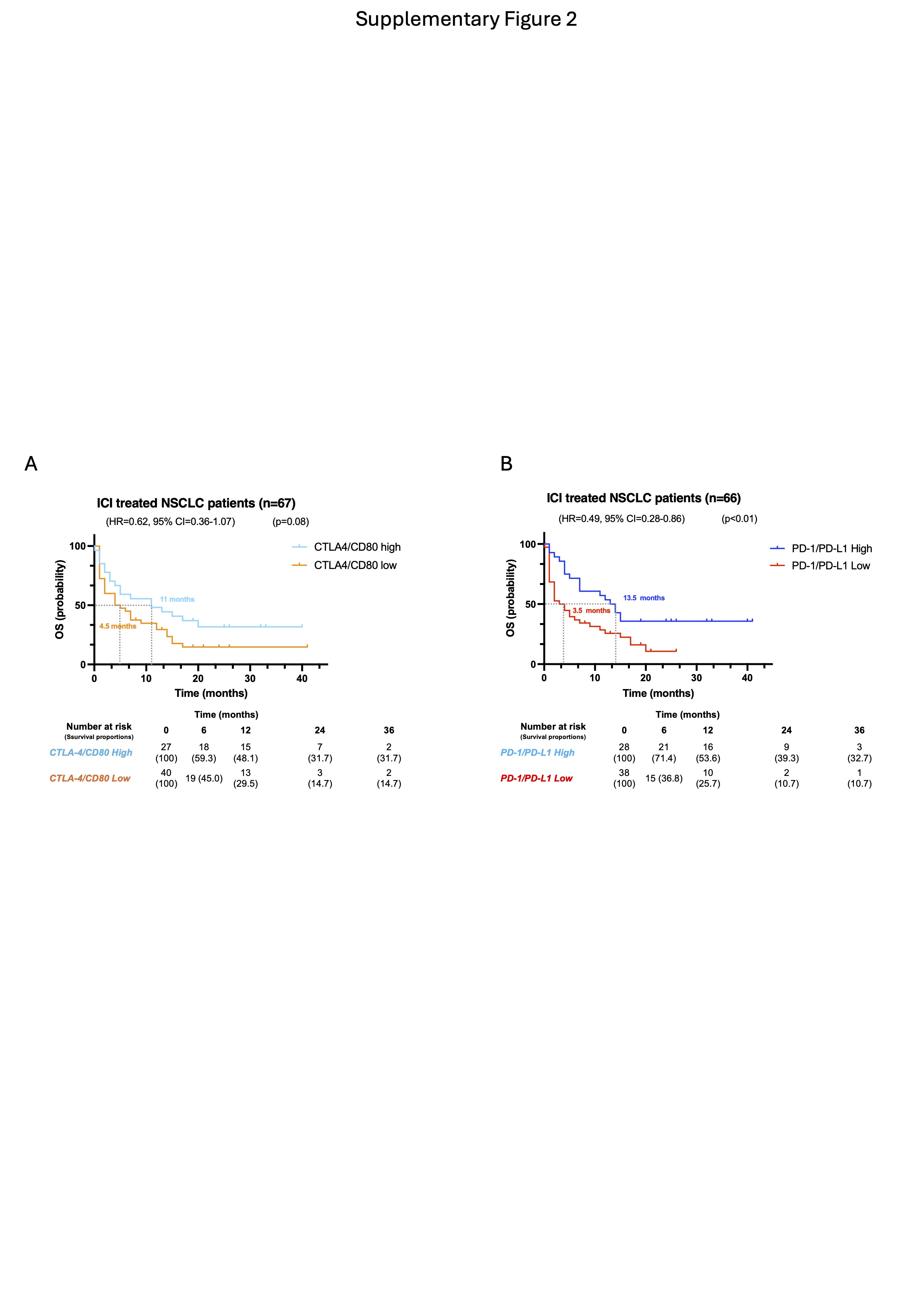

Supplement: Supplementary file 2 [file Image2.jpeg]

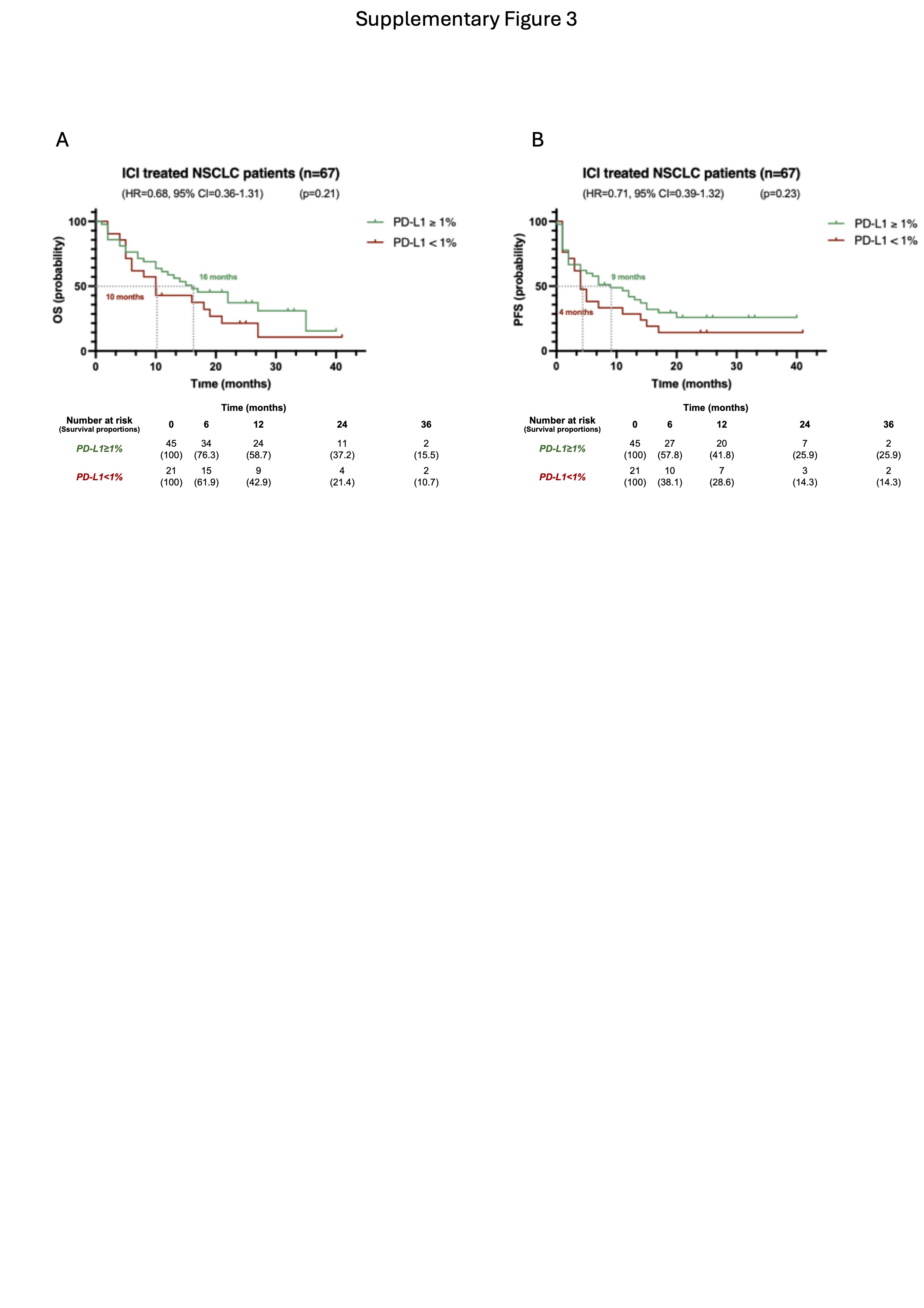

Supplement: Supplementary file 3 [file Image3.jpeg]

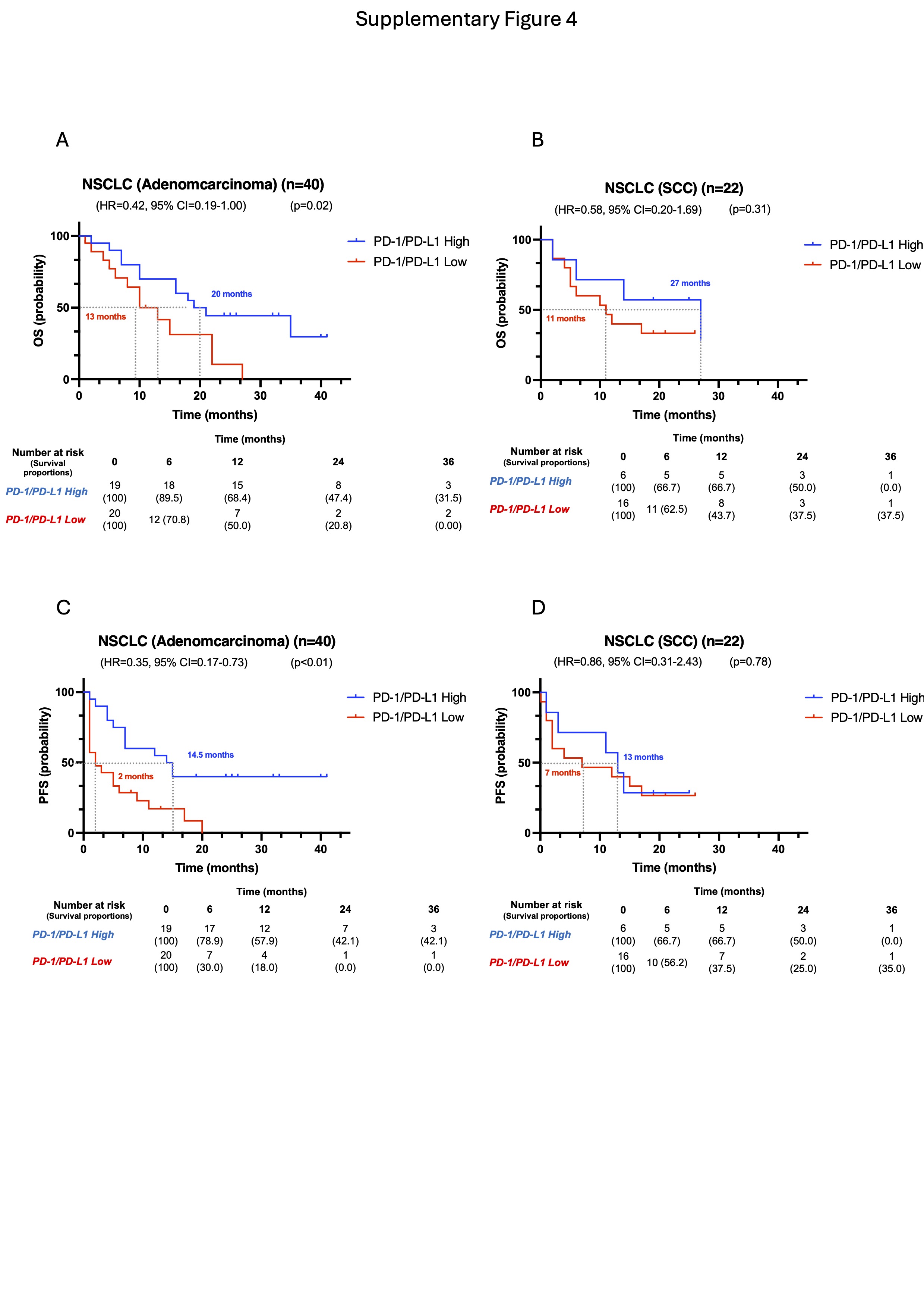

Supplement: Supplementary file 4 [file Image4.jpeg]

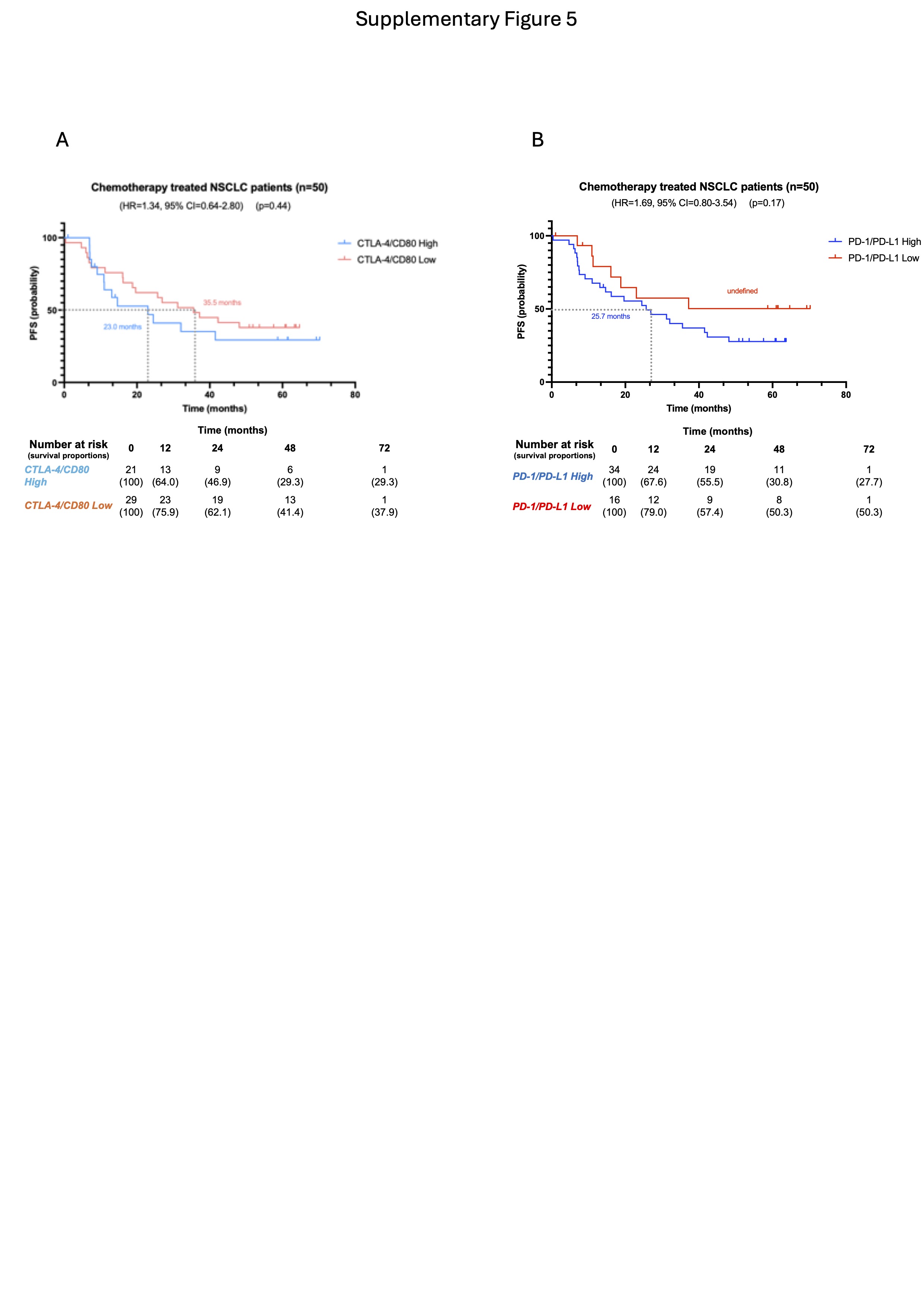

Supplement: Supplementary file 5 [file Image5.jpeg]

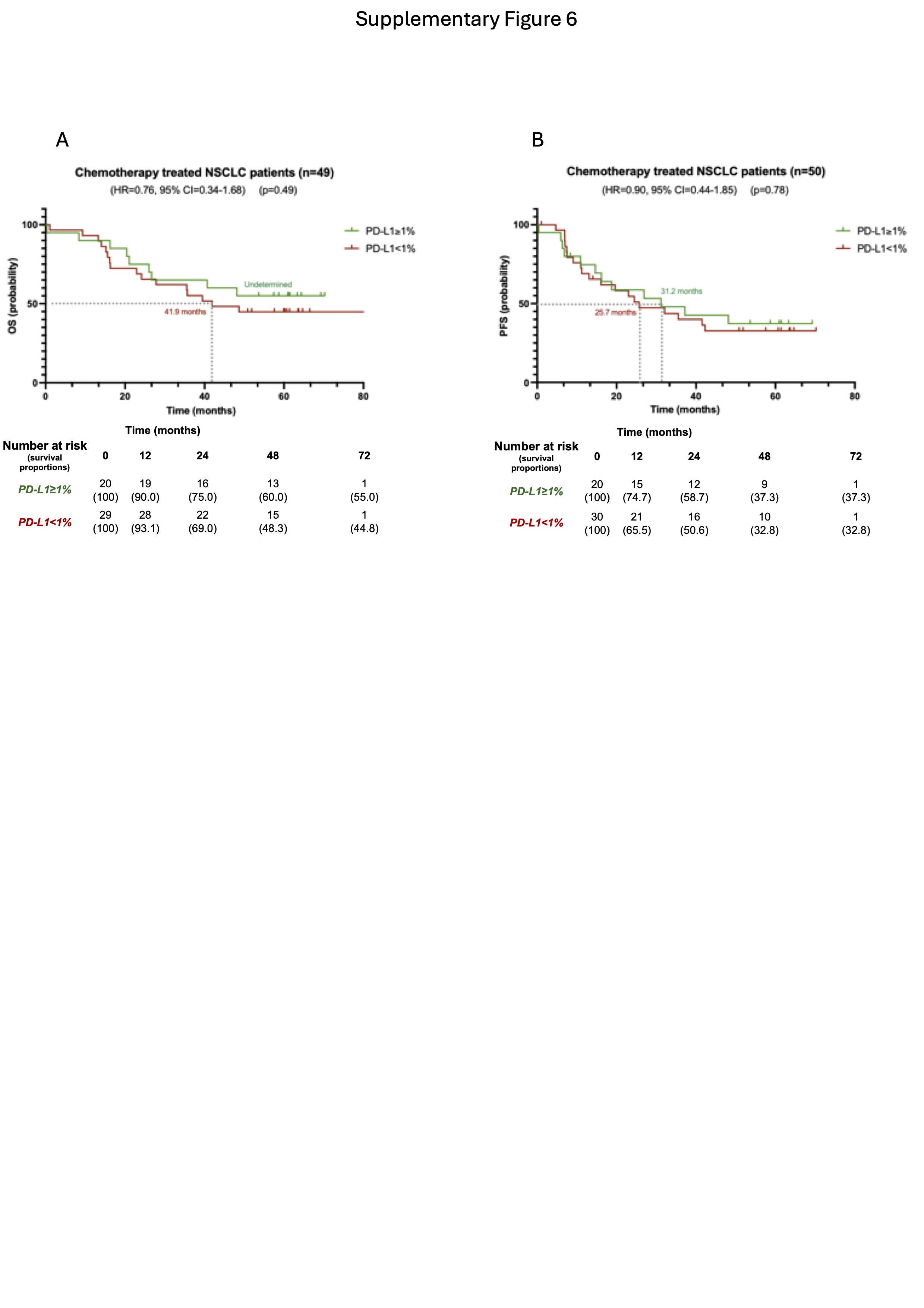

Supplement: Supplementary file 6 [file Image6.jpeg]

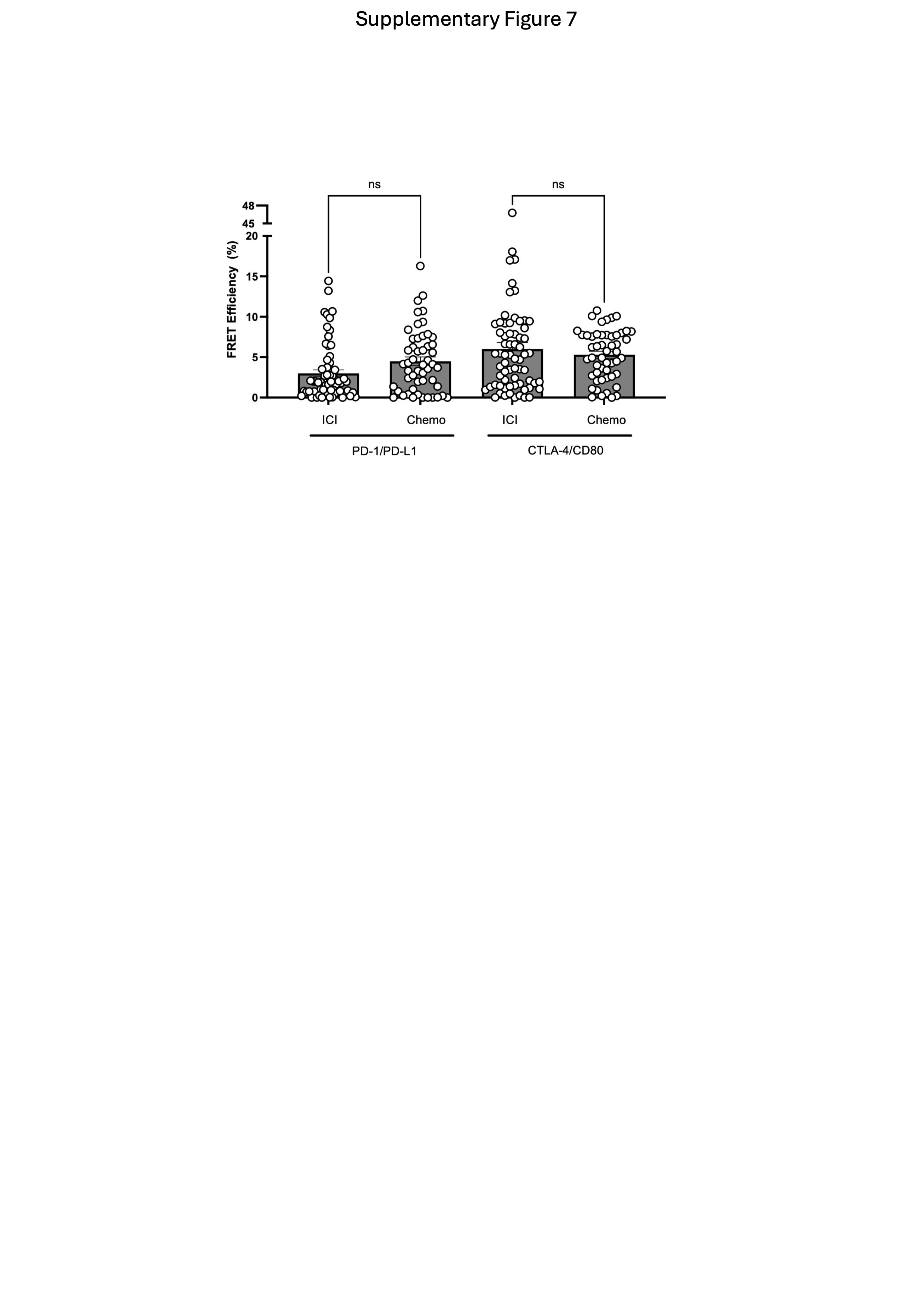

Supplement: Supplementary file 7 [file Image7.jpeg]

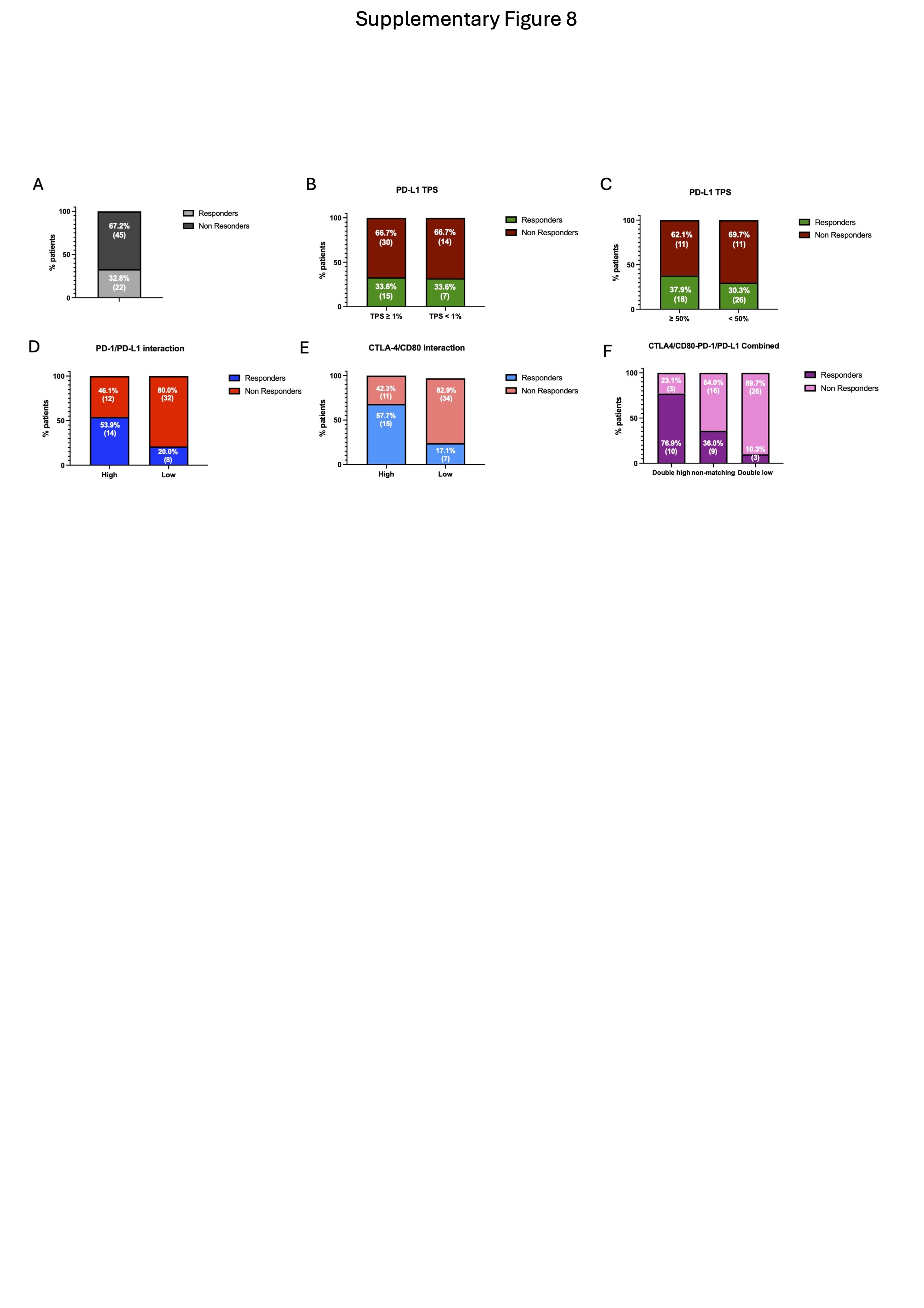

Supplement: Supplementary file 8 [file Image8.jpeg]

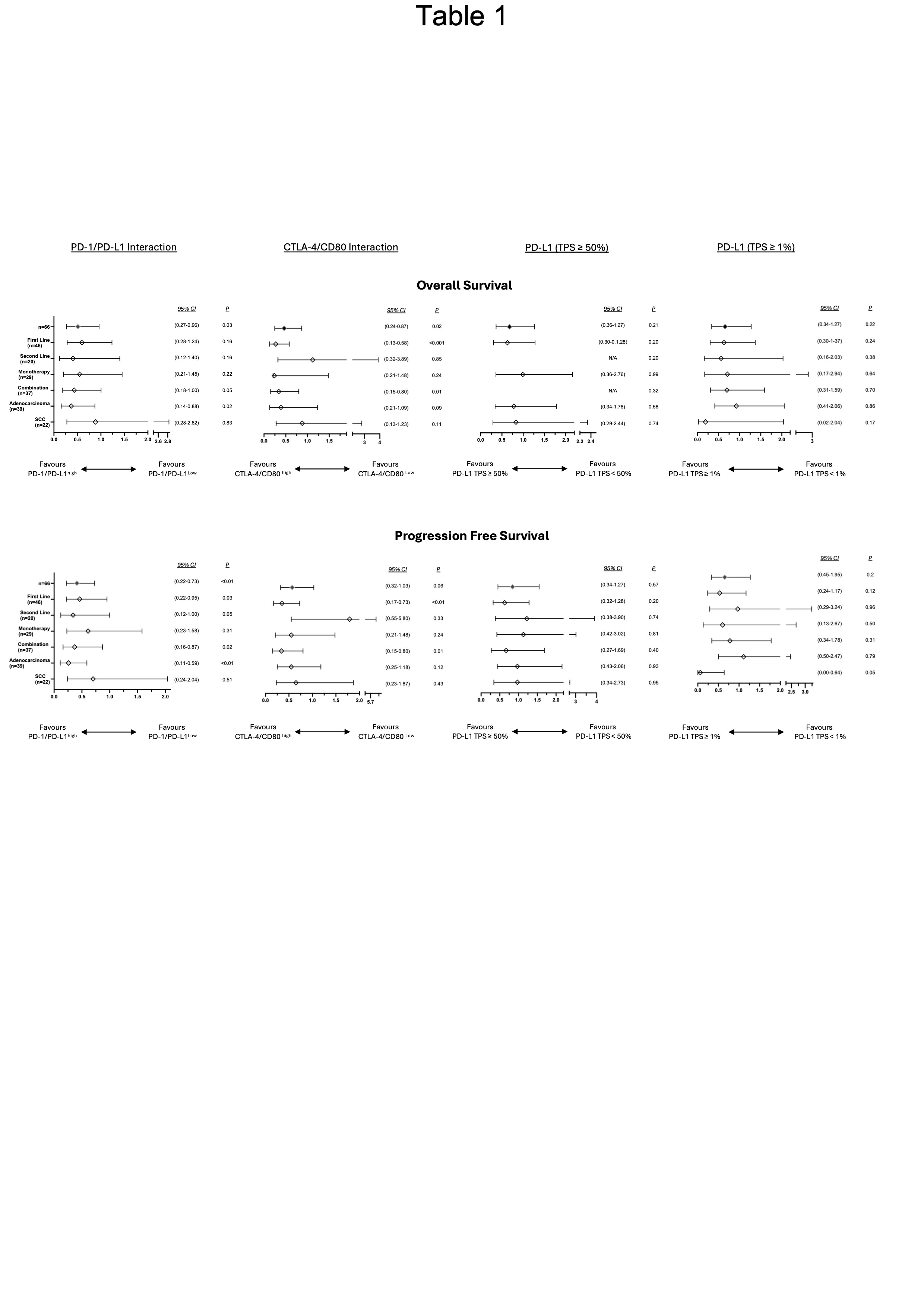

Supplement: Supplementary file 9 [file Supplementaryfile1.jpg]

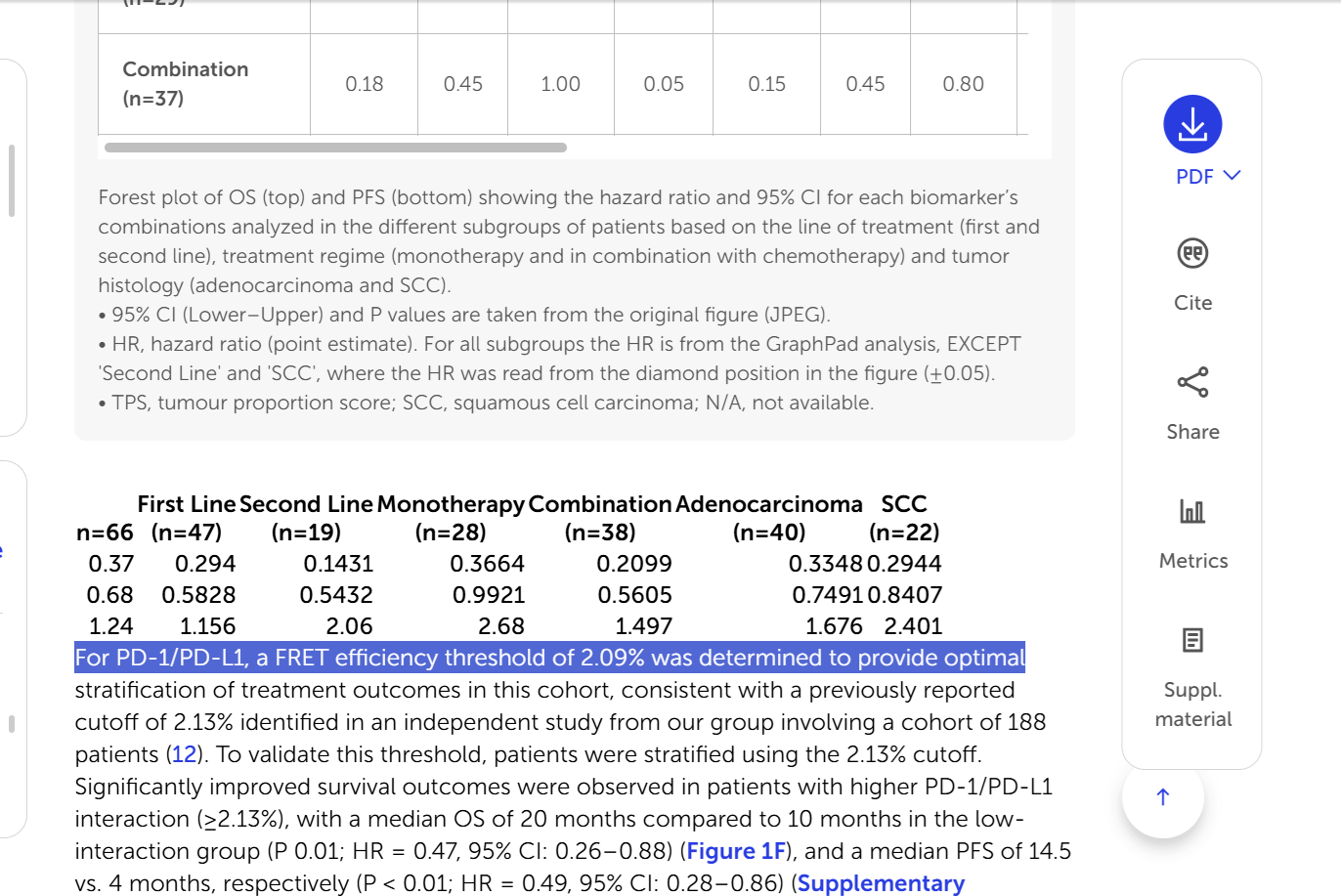

Supplement: Supplementary file 10 [file Supplementaryfile3.png]
